# Opportunistic Bacteria Dominate the Soil Microbiome Response to Phenanthrene in a Microcosm-Based Study

**DOI:** 10.3389/fmicb.2018.02815

**Published:** 2018-11-21

**Authors:** Sean Storey, Mardiana Mohd Ashaari, Nicholas Clipson, Evelyn Doyle, Alexandre B. de Menezes

**Affiliations:** ^1^School of Biology and Environmental Science, University College Dublin, Dublin, Ireland; ^2^Earth Institute, University College Dublin, Dublin, Ireland; ^3^Department of Biotechnology, Kulliyah of Science, International Islamic University Malaysia, Malaysia, Malaysia; ^4^Microbiology, School of Natural Sciences, Ryan Institute, National University of Ireland, Galway, Ireland

**Keywords:** polycyclic aromatic hydrocarbons, microbiome, bioremediation, soil, phenanthrene

## Abstract

Bioremediation offers a sustainable approach for removal of polycyclic aromatic hydrocarbons (PAHs) from the environment; however, information regarding the microbial communities involved remains limited. In this study, microbial community dynamics and the abundance of the key gene (PAH-RHDα) encoding a ring hydroxylating dioxygenase involved in PAH degradation were examined during degradation of phenanthrene in a podzolic soil from the site of a former timber treatment facility. The 10,000-fold greater abundance of this gene associated with Gram-positive bacteria found in phenanthrene-amended soil compared to unamended soil indicated the likely role of Gram-positive bacteria in PAH degradation. In contrast, the abundance of the Gram-negative PAHs-RHDα gene was very low throughout the experiment. While phenanthrene induced increases in the abundance of a small number of OTUs from the Actinomycetales and Sphingomonadale, most of the remainder of the community remained stable. A single unclassified OTU from the *Micrococcaceae* family increased ~20-fold in relative abundance, reaching 32% of the total sequences in amended microcosms on day 7 of the experiment. The relative abundance of this same OTU increased 4.5-fold in unamended soils, and a similar pattern was observed for the second most abundant PAH-responsive OTU, classified into the *Sphingomonas* genus. Furthermore, the relative abundance of both of these OTUs decreased substantially between days 7 and 17 in the phenanthrene-amended and control microcosms. This suggests that their opportunistic phenotype, in addition to likely PAH-degrading ability, was determinant in the vigorous growth of dominant PAH-responsive OTUs following phenanthrene amendment. This study provides new information on the temporal response of soil microbial communities to the presence and degradation of a significant environmental pollutant, and as such has the potential to inform the design of PAH bioremediation protocols.

## Introduction

Soil pollution is a global problem; there are about 342,000 contaminated sites in Europe alone, with this number set to increase to over 500,000 by 2025 (Anon, 2012). Polycyclic aromatic hydrocarbons (PAHs) are a class of toxic chemicals, composed of at least two benzene rings arranged in various conformations (Wickliffe et al., 2014). PAHs are released to the environment upon incomplete combustion of fossil fuels and are ubiquitous in nature, with soil as their major sink (Collins et al., 2013). Due to their carcinogenic and mutagenic potential, 16 of these compounds are listed as priority pollutants by the United States Environmental Protection Agency (Wang et al., 2010).

Bacterial communities are important components of soil environments and are essential in the provision of many ecosystem services (Brussaard, 2012), including degradation of toxic chemicals such as PAHs (Fernández-Luqueño et al., 2011). However, the hydrophobicity of PAHs renders them poorly available to microorganisms, leading to their persistence in the environment (Posada-Baquero and Ortega-Calvo, 2011). A wide range of bacteria and fungi capable of degrading PAHs have been isolated from contaminated sites (for reviews see Cerniglia, 1992, 1997; Doyle et al., 2008; Ghosal et al., 2016), with *Pseudomonas, Sphingomonas*, and *Mycobacterium* spp. amongst the most frequently isolated PAH degrading bacteria (Bastiaens et al., 2000; Johnsen and Karlson, 2005). Culture independent analyses have revealed that representatives of Proteobacteria, Actinobacteria, and Firmicutes are the bacteria most likely to increase during biodegradation of PAHs and petroleum hydrocarbons (Fuentes et al., 2014).

Although bioremediation approaches have been successfully applied to PAH-contaminated soils both in the laboratory (Sayara et al., 2011) and in the field (Lors et al., 2012), the process remains poorly understood, mainly in terms of the structure and function of microbial assemblages involved (Yang et al., 2015). PAH biodegradation studies have been analyzed using different molecular techniques (Muckian et al., 2009; Lors et al., 2010; Sun et al., 2012; Festa et al., 2016; Kuppusamy et al., 2016). In general, individual molecular microbial studies of PAH degradation targeted complex bioremediation conditions. For example, Jiao et al. (2017) showed that a variety of pollutants had consistent, broadly defined effects on soil microbial taxa, mostly repressing or inducing specific microbial populations. Zhao et al. (2016) showed that *Mycobacterium* contribute ring-hydroxylating dioxygenases involved in the initial steps of fluoranthene breakdown, while a more diverse group of bacteria contributed to the metabolism of downstream fluoranthene degradation products. Kuppusamy et al. (2016) demonstrated that metal-resistant, PAH-degrading Alphaproteobacteria can persist for longer in soils contaminated with heavy metals and PAHs compared to Gram-positive bacteria such as the Actinobacteria. Certain amendments can increase bioremediation efficiency, for example Wang et al. (2016) showed that surfactants increased PAH removal from soil and boosted the abundance of the *Pseudomonas, Bacillus* and *Sphingomonas*. The inoculation of PAH-degrading consortia into polluted soil can also enhance bioremediation and boost the abundance of genes associated with formation of PAH degradation products that can be further metabolized through the tricarboxylic acid cycle (Zafra et al., 2016). Festa et al. (2016) showed that inoculation of PAH-degrading *Sphingobium* increases the degradation of phenanthrene in soil but had no effect in the degradation of PAHs in a chronically contaminated soil. Other studies found less clear relationships between soil amendments, soil microbial communities and PAH degradation. For example, Thomas and Cébron (2016) found that phenanthrene amendment in the presence of plants did not lead to greater removal of phenanthrene from bulk soil, despite increases in the abundance of PAH-ring hydroxylating genes. Similarly, Delgado-Balbuena et al. (2016) did not find strong relationships between soil microbial community structure and anthracene removal from contaminated soil when applying different remediation strategies. Although these studies offer valuable insights into the microbial molecular ecology of PAH degradation in soils, they vary substantially in their objectives, target habitat, PAH type, and experimental design. Currently, studies examining the effects of a single PAH in the native soil microbiome without the confounding effects of multiple treatments and contaminants are limited.

PAH contamination causes physiological stress on the soil microbial communities and exposure to these compounds leads to the activation of detoxification and stress resistance (de Menezes et al., 2012). Therefore, PAH exposure not only triggers the growth of PAH-degrading microorganisms, but it may also affect the ecological stability of the soil microbiome, in turn potentially affecting a range of soil processes (Griffiths and Philipot, 2013). Microbial ecosystem stability is affected by species richness, evenness, and composition, however greater biodiversity levels are not necessarily a sign of a more stable ecosystem (Griffiths and Philipot, 2013; Shade, 2017). While the intermediate disturbance hypothesis was postulated to explain the frequent observation in macro-ecology of maximal biodiversity levels at intermediate levels of disturbance, the applicability of this hypothesis to microbial ecosystems is less certain (Gibbons et al., 2016). In microbial communities, disturbance is thought to cause changes in community composition, leading to a succession of species in which opportunistic, fast growing bacteria able to take advantage of transient conditions thrive initially, followed by slower growing, resource efficient microorganisms (Sigler and Zeyer, 2004). PAH contamination in soil therefore represents an ideal opportunity to study the effects of a disturbance on microbial community composition and succession.

Bacterial diversity in soil amended with the 3-ring PAH phenanthrene was compared with that in unamended soil during the course of phenanthrene degradation using 16S rRNA gene amplicon sequencing. The soil used in this experiment had previous history of exposure to PAH pollution due to the activities of a since-closed timber treatment facility, however at the time of sampling PAH levels in this soil were similar to uncontaminated soils. Although a previous study by de Menezes et al. (2012) examined gene expression in a phenanthrene-contaminated soil, only one time-point was examined and the effect of the PAH on soil microbial community structure was not assessed. We set up two sets of microcosms in triplicate, of which one set was amended with phenanthrene and the other set used as the unamended control. These microcosms were sampled at four time points during a 17-day incubation during which time phenanthrene levels were measured and bacterial diversity analyzed by high-throughput sequencing of the 16S rRNA gene. We also assessed the potential for PAH degradation in these soils by quantifying the abundance of the gene encoding the α subunit of a PAH ring hydroxylating dioxygenase (PAH-RHDα), a key gene involved in PAH degradation. Compared to the complex experimental design of most microbiome PAH bioremediation studies, the simple experimental design used in this study allows the investigation of phenanthrene as the single treatment, without the confounding effects of multiple treatments. We observed that dominant phenanthrene-responsive bacteria showed opportunistic traits, increasing substantially in abundance in the amended samples but also to a lesser extent in the control samples, likely as a result of their capacity to adapt and grow in ecologically disturbed environments.

## Materials and methods

### Soil collection, microcosm setup, and sampling

Soil was collected from the site of a former timber treatment facility in Monard, Co. Tipperary, Ireland (52°30' N, 8°13' W). This site is elevated about 100 m above sea level and contains a mixture of gray and gray-brown podzolic soil with underlying limestone glacial till and limestone. Soil chemical analysis was carried out by ALcontrol Geochem, Dublin, Ireland, while soil particle size was determined following the method of Kettler et al. (2001). The soil had a moisture content of 18.45%, a pH of 6.58 and a total organic carbon content of 2% (w/v). The concentration of total background PAHs was 4.7 mg kg^−1^ (standard deviation 4.6%) as determined by GC-FID (Sawulski et al., 2014). The soil is a clay fine loamy drift with limestone soil, composed of 51.95% sand, 18.13% silt, and 29.91% and it is classified as luvisol.

A total of 24 microcosms were set up, 12 for each treatment (phenanthrene amendment and control) and 3 for each time point (de Menezes et al., 2012). Prior to microcosm set up, the soil was sieved to < 2 mm to remove plant matter and debris and amended with phenanthrene to a final concentration of 725 mg kg^−1^. Fifty grams of unamended or amended soil was then placed in black polyvinylchloride containers in triplicate and incubated at 22°C for the duration of the experiment (17 days). Soil moisture content was maintained at a constant level by gravimetric addition of sterile deionized water every 2–3 days. Pots were destructively sampled on days 0, 2, 7, and 17.

### Phenanthrene extraction and analysis

Phenanthrene was extracted from soil by adding acetone and hexane to soil samples and using mechanical agitation (Dean and Xiong, 2000). Phenanthrene concentration was determined by Gas Chromatography-Flame Ionization Detection as described by Storey et al. (2014).

### DNA extraction

DNA was extracted from triplicates of each treatment (3 microcosm pots were destructively harvested for each treatment at each time point) on days 0, 2, 7, and 17 of the experiment as outlined in de Menezes et al. (2012) using a modification of the phenol-chloroform method of Griffiths et al. (2000).

### Amplicon sequencing

Amplicon sequencing was carried out using the procedure detailed by Martínez et al. (2009). Briefly, the V1-V3 regions of the 16S rRNA gene were amplified using 8F-518R primers (Lane et al., 1985; Muyzer et al., 1993) which contained specific adapter sequences and unique barcodes for each sample. GENETOOLS software (Syngene, Cambridge, UK) was used for quality control of PCR reactions. Equal amounts of amplicons from each PCR reaction were pooled, run on a gel to ensure purity and DNA concentrations then measured using the Quant-iT™ PicoGreen™ dsDNA Assay Kit (Thermo Fisher, Scientific, MS, USA) and a Qubit fluorimeter (Thermo Fisher, Scientific, MS, USA). Pyrosequencing was performed using the Roche-454 Titanium platform at the University of Nebraska-Lincoln Core for Applied Genomics and Ecology. Sequence files associated with each sample were deposited in the NCBI GenBank database under the BioProject accession number PRJNA284664.

Sequences were processed using mothur v.1.31.0. with default parameters for 454-Titanium sequence processing (Schloss et al., 2009). Briefly, after removing sequence noise, sequences smaller than 200 bp, with 1 or more nucleotide ambiguities or > 8 bp long homopolymers were removed from the dataset. Sequences were aligned against the Silva reference alignment and those sequences classified as plastid, mitochondrial, archaeal, eukaryotic or unknown at the kingdom level were discarded. Chimeras were detected using the UCHIME tool built within mothur (Edgar et al., 2011) and also removed from the dataset. After quality processing the number of sequences per sample ranged from 2,572 to 11,096 sequences. Operational taxonomic units (OTUs) were generated by calculating pairwise distances and clustering sequences with a distance cutoff of 0.03. OTUs present as a single sequence read in the dataset were removed. The OTUs were classified by obtaining the consensus taxonomy in mothur using the RDP reference files and a consensus confidence threshold cutoff of 0.8 (Schloss, 2009).

Relative abundances were determined in mothur as the abundance of each OTU to the total number of sequences in a sample. For beta-diversity analyses, the number of sequences across samples was subsampled using the sub.sample command in mothur to the number of sequences present in the sample with smallest number of sequences. The microbial community composition of one sample from day two of the control microcosms was substantially different from all other samples in the dataset and was no more similar to its two biological replicates than to samples from the other treatments. We therefore chose to remove this sample from the analysis. In order to identify individual OTUs that were enriched in PAH-amended microcosms, we used DESeq2, which has the advantage of not requiring rarefaction or subsampling of sequence data, a procedure that leads to loss of valid sequence information (McMurdie and Holmes, 2013, 2014; Love et al., 2014). DESeq2 tests were performed using the Wald test, automatic filtering of low abundance OTUs, and an alpha of 0.05 and multiple testing adjustment of *p*-values. Bacterial alpha diversity (Shannon index) was also calculated in Phyloseq, and paired t-tests using Bonferroni adjusted *p*-values were carried out to determine the significance of differences in Shannon index between treatments.

### Quantitative PCR

To generate standard curves of the RHDα-GP and the PAH-RHDα-GN genes, both genes were amplified from soil using the primer sets described in Cébron et al. (2008). End-point PCRs were performed initially in 25-μl volumes containing 12.5 μl 2X PCR master mix (Promega), 2.5 pmoles each primer, 0.2 μl ultrapure BSA (50 mg ml−1, Ambion), and 10 ng DNA template. The thermocycling conditions were as follows: 95°C for 5 min (one cycle); 95°C for 30 s, 57 (GP) or 54 °C (GN) for 30 s, 72°C for 30 s (30 cycles); 72°C for 7 min. PCR products were visualized on a 1.2% (w/v) agarose gel. The resulting products were cloned using the pGEM-T Easy Vector system (Promega), and clones containing both plasmid and insert were isolated and purified using the PureYield™ Plasmid Miniprep system (Promega). Plasmid DNA was quantified using a Nanodrop™ ND-1000 Spectrophotometer (Thermo Scientific). The copy number of the PAH-RHDα-GP and the PAH-RHDα-GN gene per volume was calculated using recombinant plasmid sizes of 3307 and 3321 bp, respectively, and a mass of 1.096 × 10–21 g bp−1. Serial dilutions were used to construct standard curves.

qPCR was then carried out using the Applied Biosystems Viia 7 qPCR machine (Life Technologies, Dublin, Ireland) in MicroAmp®optical 96-well reaction plates containing 6.25 μl of 2 × KAPA SYBR® FAST qPCR master mix, 0.25 μl ROX™ low passive reference dye (Anachem, Bedfordshire, UK), 0.2 pmoles each primer and 2 ng DNA. Nuclease-free water (Sigma-Aldrich, Arklow, Ireland) was added to give a final volume of 12.5 μl. No-template controls contained 1 μl nuclease-free water (Sigma-Aldrich, Arklow, Ireland) in place of DNA template. Amplification was carried out using a modification of the method of Cébron et al. (2008) as follows: 95°C for 1 min, then 40 cycles of 95°C for 30 s, annealing temperature for 30 s, then elongation at 72°C for 30 s. SYBR® Green signal intensity was then measured during a 10 s primer dissociation step at 80°C. A melting curve was included at the end of each reaction using a temperature increment of 0.05°C s^−1^ from 51–95°C. Standard curves were included in each experiment. All analyses were performed using ABI VIIA 7 software version 1.2.2 (Life Technologies, Carlsbad, USA), Microsoft Excel 2010 and SAS version 9.1 (SAS, Cary, USA).

### Data analysis

Multivariate statistical analysis was carried out in PRIMER version 6.1.9 with PERMANOVA add-on version 1.0.1 (Primer-E Ltd., Plymouth, UK). Statistical analysis of the relative abundance of OTUs was assessed at genus level using PRIMER. Permutational multivariate analysis of variance (PERMANOVA) was performed using a Bray Curtis dissimilarity matrix in PRIMER using 9,999 unrestricted permutation of raw data. Analysis of variance (ANOVA) was carried out using SAS version 9.1. Principal coordinates analysis (PCoA) was performed using the capscale command in the vegan package in R (Oksanen et al., 2018), using square-root transformed genus-aggregated sequence abundances and a Bray-Curtis dissimilarity matrix, with overlaid arrows representing correlations > 0.4 between genera abundance profiles and PCoA ordination axes. Pearson correlation between selected OTUs and total abundance of the PAH-RHDα gene was calculated in R using the cor.test function.

## Results

### Bacterial community response to phenanthrene

The response of the indigenous soil bacterial community during phenanthrene degradation was examined using 454 amplicon sequencing. PERMANOVA analysis (Table 1) indicated that bacterial community structure changed both over time and in the presence of phenanthrene. Pairwise comparisons on bacterial community structures in phenanthrene amended soil revealed that this PAH had no significant effect on bacterial community structures on days 0 and 2, but bacterial community structures on day 7 of phenanthrene amended soil were significantly different from those on day 17. In addition, bacterial community structure in phenanthrene amended soil on days 7 and 17 were significantly different to those in the unamended control soil (*P* < 0.001; Supplementary Table 1). When temporal changes were examined, bacterial community structure was found to change significantly between each sampling day in the unamended control (*P* < 0.001), but no changes were observed in the phenanthrene-amended soil between days 0 and 2 (*P* = 0.09).

**Table 1:** PERMANOVA results for amplicon sequencing analysis of bacterial communities at different taxa levels in soil amended or unamended with phenanthrene on days 0, 2, 7, and 17.

**Taxon**	**Time**	**Phenanthrene**	Time^*^phenanthrene
Phylum	6.87^**^	12.11^**^	2.41^*^
Family	12.31^**^	10.96^**^	4.07^**^
Genus	11.29^**^	10.12^**^	4.19^**^
OTU	10.41^**^	9.17^**^	4.02^**^

*Pseudo-F values are given, with P-values in superscript*.

* *p < 0.05*;

** *p < 0.01*.

**Figure 1:**
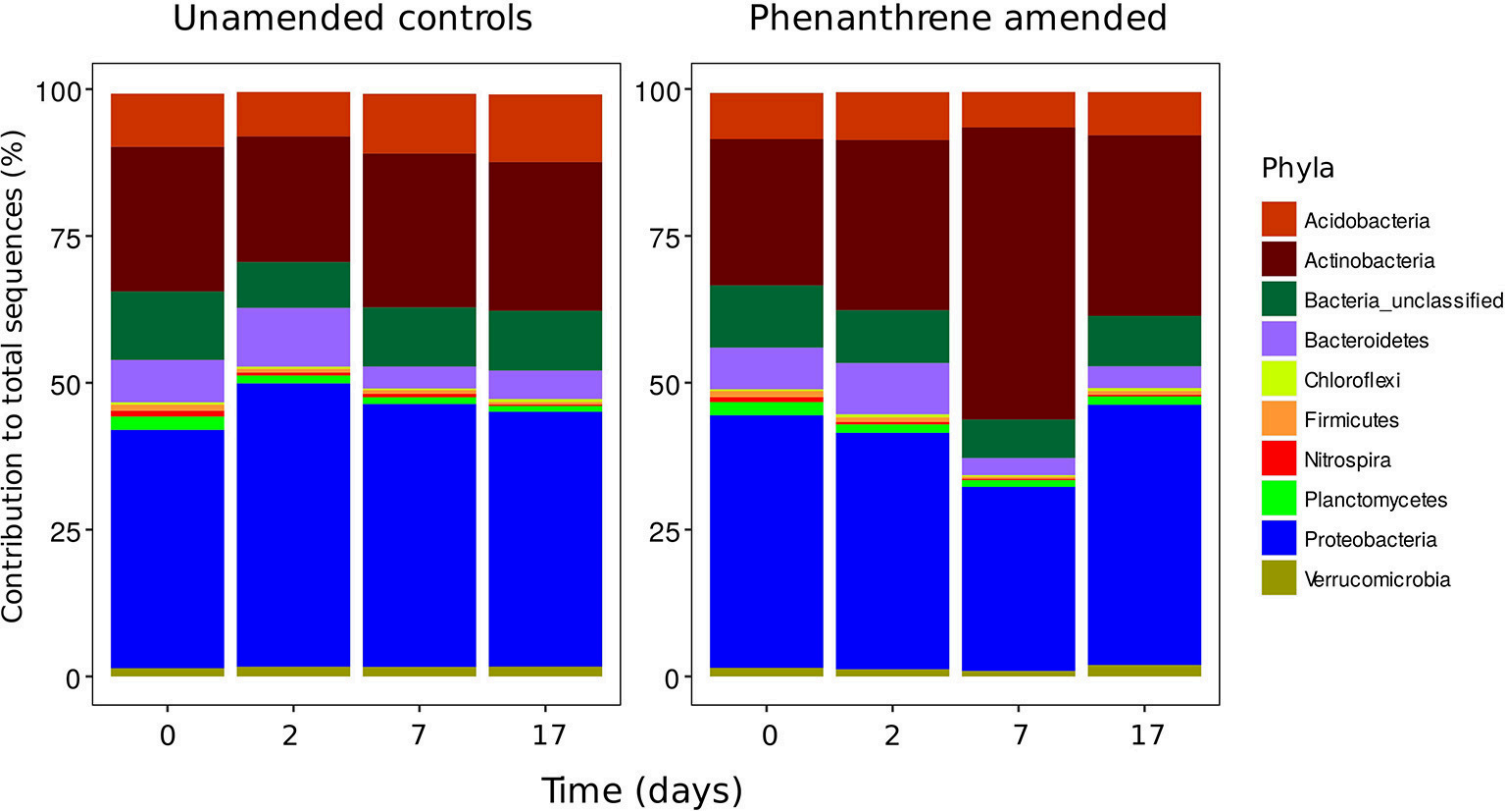
Phyla contributing >1% to the total number of sequences in unamended or phenanthrene-amended microcosm soils on days 0, 2, 7, and 17.

Bacterial diversity was similar in both unamended and phenanthrene-amended soils on day 0, with soils dominated by two phyla the Actinobacteria and the Proteobacteria (Figure 1). Although a small increase in the relative abundance of Actinobacteria and concurrent decrease in the relative abundance of the Proteobacteria was observed between days 0 and 2 in the unamended soil, these changes were not statistically significant. In the phenanthrene amended soil, degradation proceeded rapidly following a brief lag period of approximately 5 days. 252 mg kg^−1^ (about 35%) of phenanthrene added remained after 7 days, with almost complete degradation observed by day 17 (de Menezes et al., 2012). In this soil, the relative abundance of the Actinobacteria was highest on day 7, representing 50% of the total amount of 16S rRNA gene sequences present. The relative abundance of this phylum was significantly (*P* < 0.05) lower in the unamended soil at the same time point. The relative abundance of sequences belonging to the Proteobacteria decreased significantly (*P* < 0.05) between days 2 and 7. By the end of the experiment (day 17), the relative abundance of the Actinobacteria in the phenanthrene-amended soil had returned to the levels observed on day 2, whereas, the relative abundance of the Proteobacteria returned to the levels observed initially (day 0).

ANOVA analysis showed that on day 7 unclassified *Micrococcaceae* and *Microbacteriaceae* were significantly (*P* < 0.05) relatively more abundant in phenanthrene-amended soils compared to the unamended control soil (Table 2). The unclassified *Micrococcaceae* and *Microbacteriaceae* increased, respectively from 9 to 32% and from 1.4 to 3% of the total 16S rRNA gene sequences in the amended soils. The relative abundance of many genera, including a range of unclassified Bacteria belonging to the *Alphaproteobacteria, Burkholderiales*, and *Bradyrhizobium* (all members of the Proteobacteria), declined significantly (*P* < 0.05) in the presence of phenanthrene. Interestingly, many genera were not significantly affected by the presence of phenanthrene at this time point.

**Table 2:** Genera contributing >1 % to the total number of 16S rRNA genes sequences in phenanthrene-amended (PHE+) and unamended (PHE-) microcosm soils on day 7.

				**Contribution to total sequences (%)**			
**Phylum**	**Class**	**Family**	**Genus**	**PHE-**	**Standard error**	**PHE+**	**Standard error**
*Actinobacteria*	*Actinobacteria*	*Micrococcaceae*	*Micrococcaceae*_unclassified	9.35a	0.75	32.42b	1.39
*Bacteria*_unclassified	*Bacteria*_unclassified	Bacteria_unclassified	*Bacteria*_unclassified	6.73a	0.32	4.66b	0.09
*Acidobacteria*	*Acidobacteria*	*Acidobacteria Gp6* incertae sedis	*Acidobacteria Gp6* incertae sedis	7.19a	0.44	3.83b	0.05
*Actinobacteria*	*Actinobacteria*	*Actinomycetales*_unclassified	*Actinomycetales*_unclassified	4.09a	0.37	3.80a	0.07
*Actinobacteria*	*Actinobacteria*	*Actinobacteria*_unclassified	*Actinobacteria*_unclassified	4.47a	0.25	3.48a	0.23
*Proteobacteria*	*Alphaproteobacteria*	*Alphaproteobacteria*_unclassified	*Alphaproteobacteria*_unclassified	4.56a	0.36	3.32b	0.46
*Actinobacteria*	*Actinobacteria*	*Microbacteriaceae*	*Microbacteriaceae*_unclassified	1.42a	0.15	3.04b	0.31
*Proteobacteria*	*Alphaproteobacteria*	*Sphingomonadaceae*	*Sphingomonas*	3.19a	0.06	2.90a	0.22
*Actinobacteria*	*Actinobacteria*	*Acidimicrobiaceae*	*Ilumatobacter*	2.65a	0.06	2.35b	0.23
*Proteobacteria*	*Alphaproteobacteria*	*Rhizobiales*_unclassified	*Rhizobiales*_unclassified	3.95a	0.09	2.30b	0.10
*Proteobacteria*	*Betaproteobacteria*	*Comamonadaceae*	*Comamonadaceae*_unclassified	1.45a	0.06	1.69b	0.03
*Proteobacteria*	*Gammaproteobacteria*	*Gammaproteobacteria*_unclassified	*Gammaproteobacteria*_unclassified	2.90a	0.26	1.56b	0.07
*Proteobacteria*	*Betaproteobacteria*	*Betaproteobacteria*_unclassified	*Betaproteobacteria*_unclassified	3.23a	0.54	1.49b	0.13
*Bacteroidetes*	*Flavobacteria*	*Flavobacteriaceae*	*Flavobacterium*	1.00a	0.11	1.34a	0.26
*Actinobacteria*	*Actinobacteria*	*Nocardiaceae*	*Nocardioides*	0.98a	0.06	1.35a	0.09
*Acidobacteria*	*Acidobacteria*	*Acidobacteria Gp16* incertae sedis	*Acidobacteria Gp16* incertae sedis	1.69a	0.33	1.33a	0.15
*Proteobacteria*	*Gammaproteobacteria*	*Xanthomonadaceae*	*Lysobacter*	0.87a	0.10	1.23a	0.20
*Proteobacteria*	*Alphaproteobacteria*	*Bradyrhizobiaceae*	*Bradyrhizobium*	2.65a	0.02	1.22b	0.07
*Proteobacteria*	*Betaproteobacteria*	*Burkholderiales*_unclassified	*Burkholderiales*_unclassified	1.54a	0.10	1.06b	0.07
*Proteobacteria*	*Alphaproteobacteria*	*Phyllobacteriaceae*	*Phyllobacteriaceae*_unclassified	1.53a	0.12	1.05b	0.08

*Each measurement represents the mean of three replicate samples. Genera are presented in order of abundance in phenanthrene-amended soils. Means not sharing the same letter within a row are statistically different (p < 0.05)*.

**Figure 2:**
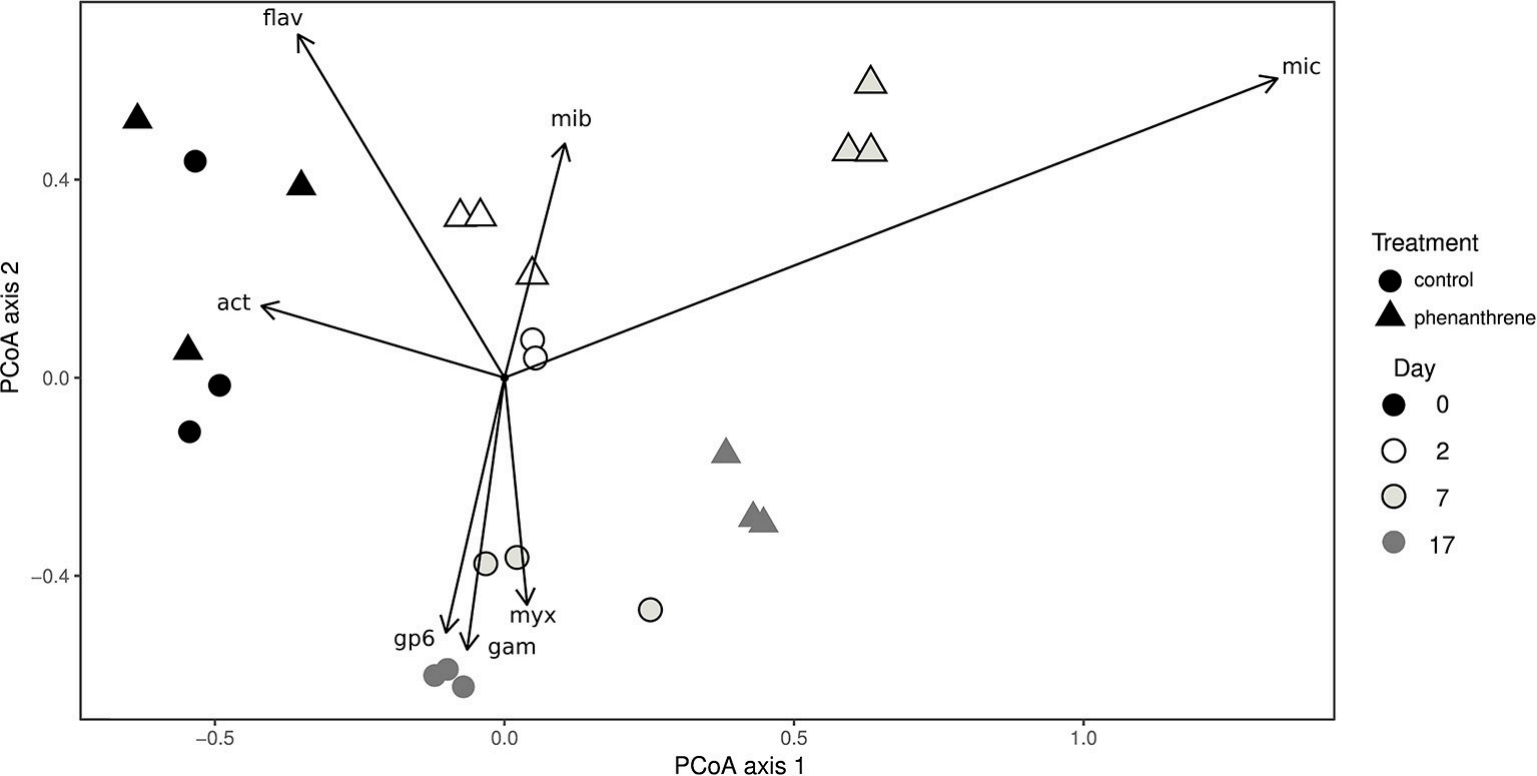
Principal coordinates analysis (PCoA) of bacterial diversity at genus level in soil amended (triangles) or unamended (circles) with phenanthrene on days 0, 2, 7, and 17. mic, *Micrococcaceae*_unclassified; mib; *Microbacteriaceae*_unclassified; act, *Actinobacteria*_unclassified; Gp6, *Acidobacteria* Gp6 incertae sedis; fla, *Flavobacterium*; gam, *Gammaproteobacteria*_unclassified; myx, *Myxococcales_*unclassified. Arrows represent correlations > 0.4 between genera and PCoA axes.

Principal coordinates analysis (PCoA) was used to correlate changes in community structure at genus level with the presence or absence of phenanthrene (Figure 2). The genus *Flavobacterium* and unclassified *Actinobacteria* were correlated with day 0 samples for both control and phenanthrene-amended samples. Two days after the start of the experiment, control and phenanthrene-amended samples were not forming clearly distinct clusters, however the unclassified *Microbacteriaceae* correlated more strongly with day 2 phenanthrene-amended samples. On day 7, control and phenanthrene-amended samples can be seen to be well separated, with the unclassified *Micrococcaceae* clearly correlating with the phenanthrene-amended samples, while day 7 and 17 control samples correlated primarily with the unclassified *Myxococcales*, the *Acidobacteria* Gp6 and unclassified *Gammaproteobacteria*. On day 17, phenanthrene-amended samples cluster more closely with the day 7 and 17 control samples.

Although ANOVA showed which bacterial genera were more abundant at each treatment, it provided limited insight into changes in individual OTUs. DESeq2 analysis, which is a more appropriate statistical test for microbiome data than ANOVA, was therefore carried out to compare the differential abundance of individual OTUs in phenanthrene-amended to unamended soils at the same time points. No significantly differentially abundant OTUs were detected on either day 0 or day 2. However, on day 7, several OTUs classified to the Actinomycetales order, including *Mycobacterium* and unclassified *Micrococcaceae* were significantly more abundant in phenanthrene-amended soil than in its unamended counterpart (Table 2). One *Mycobacterium* OTU (OTU 82) was almost 200-fold more abundant on day 17 in phenanthrene-amended compared to unamended soil. OTUs classified to several other taxa, including *Sphingomonas*, unclassified *Microbacteraceae* and *Pseudoxanthomonas* were also significantly more abundant in the phenanthrene-amended soil compared to the unamended control soil. Interestingly, few OTUs responded negatively to phenanthrene, one unclassified OTU of the Burkholderiales and an unclassified Actinomycetales OTU were significantly less abundant in the phenanthrene-amended soil on day 17 of the experiment (Table 2).

**Figure 3:**
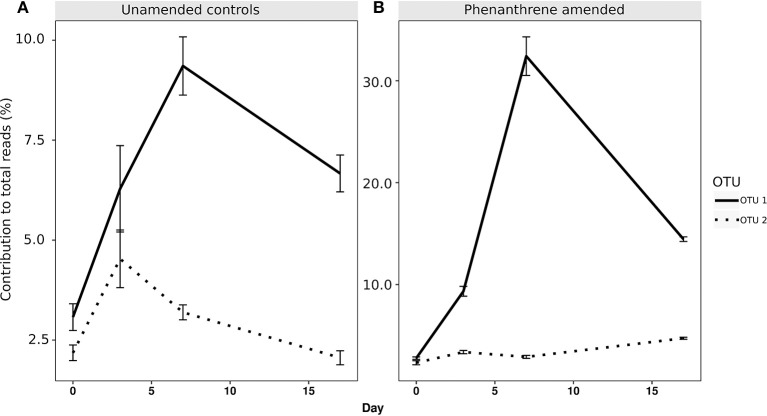
Percentage relative abundance of the two most abundant PAH-responsive OTUs in **(A)** unamended and **(B)** phenanthrene-amended microcosm soils on days 0, 2, 7, and 17. OTU 1, unclassified *Micrococcaceae*; OTU 2, *Sphingomonas* sp.

**Figure 4:**
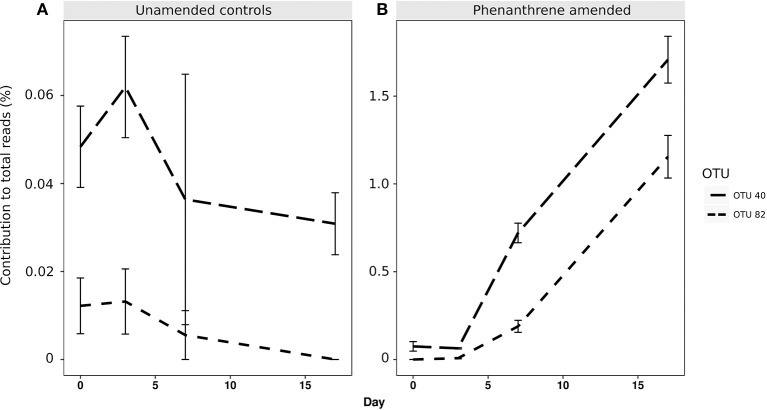
Percentage relative abundance of PAH-responsive OTUs classified to Actinomycetales (OTU 40) and *Mycobacterium* sp. (OTU 82) in **(A)** unamended and **(B)** phenanthrene-amended microcosm soils on days 0, 2, 7, and 17.

When analyzing the relative abundance of those OTUs that were found to be significantly more abundant in the amended microcosms at any time point (phenanthrene-responsive OTUs), one single OTU classified to the Micrococcaceae family (OTU 1) increased in relative abundance from approximately 2.5% (day 0) to 32% (day 7) in the phenanthrene-amended microcosms, while in the unamended microcosms OTU 1 abundance also increased on between days 0 and 7 but to a lesser extent (10% of total sequences) (Figure 3). The second most relatively abundant phenanthrene-responsive OTU (OTU 2, *Sphingomonas* sp.) increased from ~2–5% of the total sequences in the amended microcosms toward the end of the experiment, while in the unamended microcosms OTU 2 increased in abundance earlier in the experiment at day 2 to approximately 5% followed by a decline to 2.5 % of total sequences (Figure 3). It is noteworthy that both dominant phenanthrene-responsive OTUs increased in relative abundance in the unamended controls from day 0 and 2. The relative abundance of two other phenanthrene-responsive actinobacterial OTUs (OTU 40, unclassified Actinomycetales, and OTU 82, *Mycobacterium* sp.) increased in relative abundance from days 7 to 17 in the amended microcosms however in the controls their abundance decreased in this period (Figure 4).

**Figure 5:**
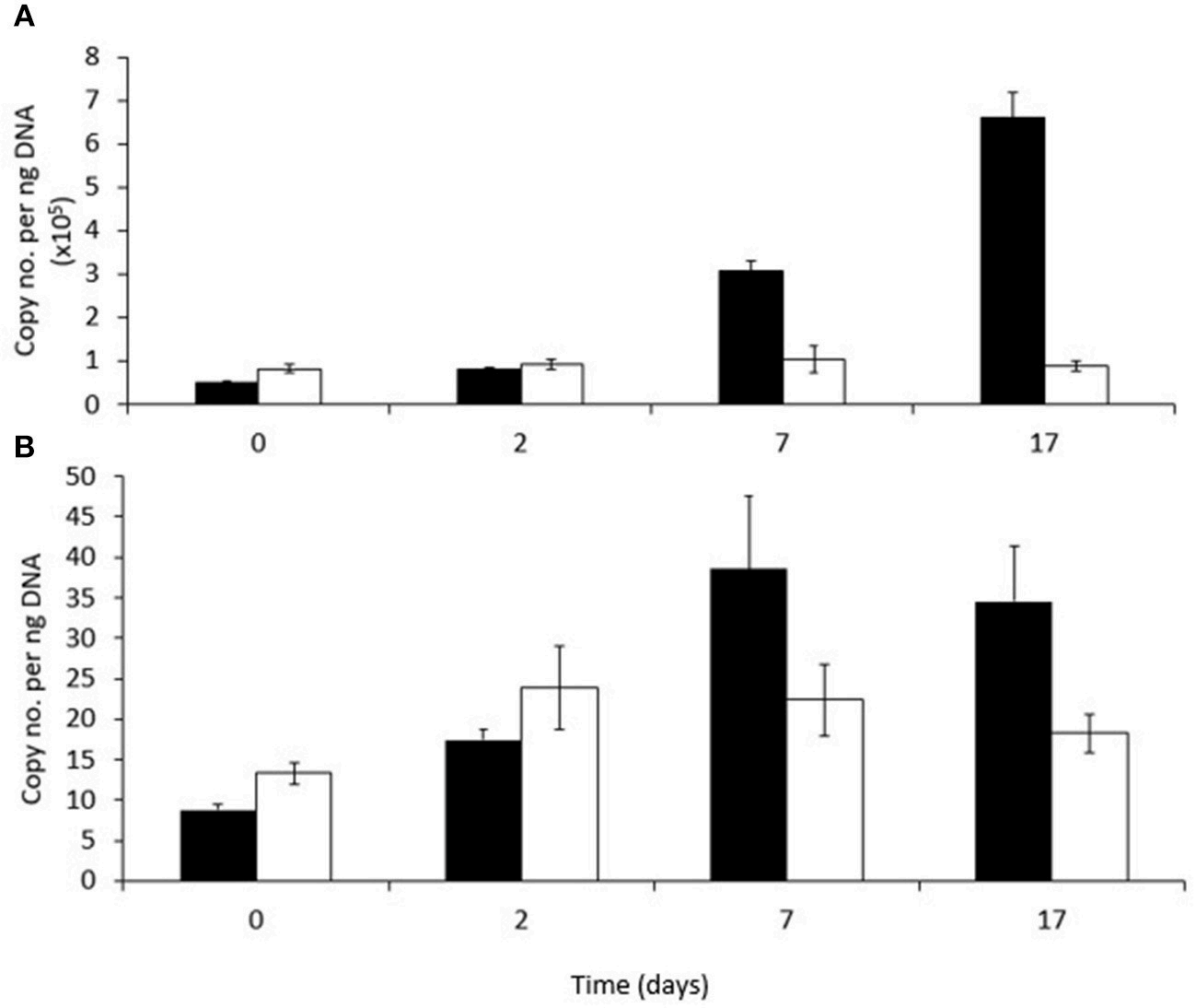
Abundance of **(A)** RHDα-GP and **(B)** RHDα-GN genes in phenanthrene-amended (black bars) or unamended (white bars) in microcosm soils on days 0, 2, 7, and 17. Each bar represents the mean of three replicates.

The Shannon index of the bacterial community did not change over the course of the experiment in the unamended control microcosms. However, it was significantly lower in the phenanthrene-amended microcosms on day 7 compared to days 0 (adjusted *p*-value < 0.001), 2 (adjusted *p*-value < 0.001), and 17 (adjusted *p-*value < 0.05; Figure 5).

### Abundance of RHDα-GP and RHDα-GN

**Figure 6:**
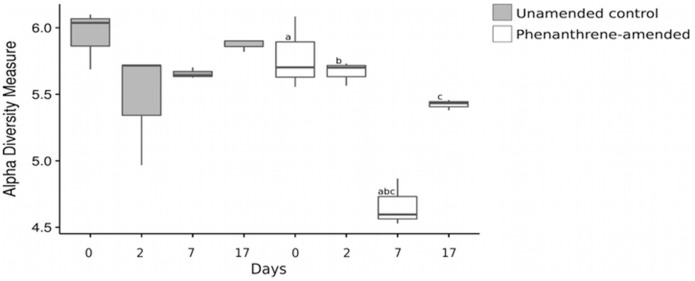
Shannon index of the bacterial community at all-time points. Shared letters represent significant adjusted *p*-values determined by pairwise *t*-tests. The lines extend to the largest value observed.

The abundance of a gene (PAH-RHDα) encoding the alpha subunit of a ring hydroxylating dioxygenase involved in the first step of PAH degradation by either Gram-positive (RHDα-GP) or Gram-negative (RHDα-GN) bacteria in both amended and unamended soils is shown in Figure 6, respectively. The abundance of this gene associated with Gram-positive bacteria (RHDα-GP) was significantly higher (between 10^3^ and 10^4^ times higher) in both unamended and phenanthrene-amended soils than the gene associated with Gram-negative bacteria (RHDα-GN) at all-time points. The relative abundance of RHDα-GP changed over time and in response to the presence of phenanthrene (*P* < 0.001). Abundance increased significantly between days 2 and 7, and again between days 7 and 17 in phenanthrene-amended samples. Abundance of RHDα-GP did not change significantly in unamended soil remaining at about 100,000 copies ng^−1^ soil throughout. RHDα-GN abundance did not change significantly either over time, or in response to phenanthrene amendment.

Analysis of the Pearson's correlation between RHDα-GP and the abundance of OTUs 40, 82, 1, and 2 showed that OTUs 40 and 82 were more strongly correlated with this gene (Pearson correlation coefficients of 0.99, *p* = 1.57^−12^ and 0.94, *p* = 2.94^−06^ for OTUs 40 and 82, respectively), compared to OTUs 1 and 2 (Pearson correlation coefficients of 0.38, *p* = 0.22 and 0.80, *p* value = 0.001 for OTUs 1 and 2, respectively).

## Discussion

PAH degradation has been shown to depend on the number of aromatic rings present, with the lower molecular weight PAHs (four or less aromatic rings) more susceptible to degradation than high molecular weight PAHs (Cerniglia, 1992). The rapid rate of degradation of the 3-ring PAH, phenanthrene, observed in this study was therefore not surprising, with similar results reported elsewhere. Sawulski et al. (2014) reported that 95% of phenanthrene (200 mg kg^−1^) was removed from a soil microcosm within two days. On day 2 of the experiment, the absence of a more pronounced phenanthrene toxic effect on the bacterial community, as indicated by the relatively small change in the relative abundances of the main bacterial phyla present, may be a result of previous exposure of these soils to creosote at the former timber treatment facility. Prior exposure to recalcitrant pollutants such as PAHs has been shown to impact soil microbial community structures (Johnsen and Karlson, 2005; Bargiela et al., 2015). Similarly, the fast rates of phenanthrene degradation observed on day 7 of the experiment are most likely a result of the prior exposure of this soil to PAHs. The constant leakage of PAHs into the soil from timber treatment may have stimulated any PAH degrading bacteria present or left a “seed” population of PAH-degrading bacteria capable of fast response to new pulses of PAH contamination. The greater phenanthrene degradation rate on day 7 coincided with a clear shift in bacterial community composition, indicating that phenanthrene degraders may have adapted and metabolized this compound. Actinobacteria and Proteobacteria are frequently observed as dominant phyla in soil (Janssen, 2006; Delgado-Baquerizo et al., 2016; Fierer, 2017) and representatives of both phyla have previously been associated with PAH degradation in soil (Mukherjee et al., 2014). In this study, both the bacterial diversity data and the abundance of the Gram-positive PAH-RHDα genes suggest that the Actinobacteria were the main contributors to phenanthrene degradation. This is supported by the previous study by de Menezes et al. (2012), which reported a significant increase in transcripts associated with dioxygenases from Actinobacteria but few from the Proteobacteria in the same soil on day 7.

The higher relative abundance of Actinobacteria in PAH amended soil appears to have been driven largely by an increase in the abundance of a single unclassified OTU from the family *Micrococcaceae* (OTU 1). Members of the *Micrococcaceae* such as *Arthrobacter phenanthrneivorans* (Kallimanis et al., 2009) and *Arthrobacter oxydans* (Thion et al., 2013) have been associated with PAH degradation, both in pure culture and in the environment (Aryal and Kyriakides-Liakopoulou, 2013). Although considerably less relatively abundant than the *Micrococcaceae*, unclassified *Microbacteriaceae* were also significantly more abundant in phenanthrene-amended soil, and this group has also been associated with PAH degradation in soil (Jacques et al., 2008; Jie et al., 2011). Although, based on relative abundance, the Proteobacteria appeared to play a smaller role in PAH-degradation in this study compared to the Actinobacteria, six OTUs classified to this phylum were significantly more abundant on days 7 and 17 in the contaminated soils compared to the controls, and this included the second most abundant phenanthrene-responsive OTU, which was classified to the *Sphingomonas* genus, often associated to PAH contamination (Leys et al., 2004). Both OTU 1 and OTU 2 were already amongst the most abundant in the test soils at the start of the experiment, which might have given them competitive advantage against other potential PAH-degrading taxa (O'Malley et al., 2010). Their original dominance in these soils may be connected to the previous history of PAH contamination of the source soil due to timber treatment, which is supported by the presence of PAH-RHDα genes in the unamended soil (Cerniglia, 1992; Johnsen and Karlson, 2005).

The involvement of OTU1 in phenanthrene degradation was suggested by the results of DeSeq2 analysis which demonstrated that the relative abundance of this OTU increased substantially at the same time as the PAH was being removed from soil. However, the relative abundance of OTU1 does not directly mirror the total abundance of the Gram-positive PAH-RHDα gene (Figures 3, 5). This discrepancy may be due to changes in the total abundance of the remainder of the bacterial community in these soils, or due to this OTU expressing a PAH-RHDα gene which is not amplified by the qPCR primers used. A further possibility is that this OTU was not directly involved in the phenanthrene ring hydrolysis, but that it utilized its degradation products generated by other members of the microbial community (Festa et al., 2013). The relative abundance of two other actinobacterial OTUs (OTU 40, unclassified Actinomycetales and OTU 82, *Mycobacterium* sp.) showed stronger correlations with the total abundance of the PAH-RHDα gene than OTUs 1 and 2, indicating that OTUs 40 and 82 may therefore have been the primary taxa involved in the initial stages of phenanthrene degradation. Actinobacteria have been reported to be the primary taxa involved in PAH degradation (Uyttebroek et al., 2006; Cébron et al., 2008; Marcos et al., 2009) and a metatranscriptomic analysis of one time point during PAH degradation in soil reported that transcripts of PAH hydroxylase genes present were primarily from the Actinobacteria (de Menezes et al., 2012). The increase in the abundance of some Gram-negative OTUs of taxa associated with PAH degradation (e.g., *Sphingomonas*) without a concurrent increase in the abundance of the Gram-negative PAH-RHDα gene could also be explained by cross feeding between ring-hydroxylating bacteria and other bacteria capable of utilizing phenanthrene breakdown products (McDonald et al., 2005).

The considerable decrease in the relative abundance of OTU 1 and the concurrent rise in the relative abundance of the Proteobacteria between days 7 and 17 in the phenanthrene-amended microcosms indicate that the community was returning to its original composition. This conclusion is further supported by the closer proximity of phenanthrene-amended day 17 samples to day 0 and 2 sample data points from phenanthrene-amended and control samples in the PCoA plot. The return of the bacterial community to its original composition demonstrates the resilience of the bacteria to the impact of phenanthrene, which is likely due to their previous exposure to PAHs in the original site from where the soil was sourced, as discussed above. Bacterial communities have been reported to recover from exposure to pollutants such as PAHs, but the length of time this recovery may take will depend on a range of factors including concentration and type of pollutant (Bordenave et al., 2007; Rodriguez et al., 2015). It may be that the growth of OTUs 1, 40, and 82 was key to the resilience of the overall bacterial community to phenanthrene on account of their possible role in removing this pollutant from the soil. With most of phenanthrene exhausted from the soil, OTU 1 may have lost its competitive advantage, and other microorganisms were then able to grow and return to their original relative abundances.

Interestingly, the same unclassified *Micrococcaceae* and *Sphingomonas* OTUs that responded positively to phenanthrene amendment also increased in relative abundance to a lesser extent in the control microcosms, following similar abundance temporal patterns as in the amended microcosms. The increase in abundance of the two dominant phenanthrene-responsive OTUs in both the PAH amended and control microcosms suggests that the ability to withstand PAH toxicity and use these compounds as growth substrates may be related to their general ecological opportunism. Although widely used to generate useful information, microcosm-based studies have several limitations (Carpenter, 1996). Harvesting of soil and set-up of microcosms can introduce oxygen and affect soil structure, which are known to affect microbial communities (Fierer et al., 2003). Opportunistic bacteria are often the first to colonize a habitat following a disturbance and show adaptations such as fast growth on limiting substrates and high tolerance to environmental stress (Sigler et al., 2002; Sigler and Zeyer, 2004; Fierer et al., 2010; Lozupone et al., 2012). It would appear that disturbance-driven ecological succession may have occurred in the phenanthrene-amended and to a lesser extent in the control soil microbiome in this experiment (Sigler and Zeyer, 2004), however the presence of phenanthrene enhanced the dominance of these opportunistic strains. The presence of an opportunistic phenotype is supported by results from the metatranscriptomic study undertaken on the same soils. Transcripts classified as heat shock proteins from Actinobacteria and Alphaproteobacteria were observed to increase when phenanthrene was added to the soil (de Menezes et al., 2012) and similar genes were identified as a genetic signature of gut early-colonizing opportunistic bacteria by Lozupone et al. (2012).

The growth and decline of the dominant PAH-responsive bacteria in the current study decreased community evenness, in agreement with the study of Thomas and Cébron (2016) who observed a decrease in bacterial community evenness two days following phenanthrene soil amendment. However, relationships between microbial evenness (Shannon diversity) and disturbance vary depending on the frequency, intensity and type of disturbance (Gibbons et al., 2016). These relationships were not tested specifically in the current experiment and warrant further investigation.

## Conclusion

The presence of phenanthrene led to significant shifts in the structure of soil bacterial communities, and this change was dominated by an increase in the relative abundance of a small number of opportunistic taxa. The prior exposure to pollution of the soil used in this experiment may have played a role in conditioning the soil community for fast responses to pulses of PAH contamination through the growth of opportunistic, fast-growing PAH-degrading bacteria mainly from the Actinobacteria phylum. The fact that 8 out of the13 PAH-responsive bacterial OTUs in this experiment were not classified to genus level (Table 3) highlights the need to better characterize uncultured soil microorganisms which have beneficial roles in bioremediation. Finally, the recognition that the soil bacterial community's response to phenanthrene contamination is at least in some cases restricted to a small number of bacterial taxa may simplify the ecological modeling and design of PAH-remediation strategies.

**Table 3:** Results from differential abundance analysis using DESeq2 (alpha = 0.05) showing the number of OTUs that are significant more abundant in either phenanthrene-amended or unamended samples at days 7 and 17 of the experiment.

**Day**	**OTU**	**Class**	**Genus**	**Average abundance of differentially abundant OTUs**	
				**Control**	**Phenanthrene**
7	OTU 1	Actinobacteria	Unclassified Micrococcaceae	9.35	32.42
	OTU 7	Actinobacteria	Unclassified Microbacteriaceae	0.85	1.83
	OTU 25	Actinobacteria	Unclassified Microbacteriaceae	0.47	0.90
	OTU 40	Actinobacteria	Unclassified Actinomycetales	0.04	0.72
	OTU 82	Actinobacteria	*Mycobacterium*	0.01	0.19
	OTU 172	Actinobacteria	Unclassified Microbacteriaceae	0.06	0.07
	OTU 187	Gammaproteobacteria	*Cellvibrio*	0.01	0.19
	OTU 320	Betaproteobacteria	Hydrogenophaga	0.00	0.14
17	OTU 1	Actinobacteria	Unclassified Micrococcaceae	6.67	14.45
	OTU 2	Alphaproteobacteria	*Sphingomonas*	2.06	4.72
	OTU 7	Actinobacteria	Unclassified Microbacteriaceae	0.68	1.31
	OTU 24	Betaproteobacteria	Unclassified Comamonadaceae	0.65	1.43
	OTU 25	Actinobacteria	Unclassified Microbacteriaceae	0.36	0.88
	OTU 32	Betaproteobacteria	Unclassified Burkholderiales	0.71	0.26
	OTU 40	Actinobacteria	Unclassified Actinomycetales	0.03	1.71
	OTU 68	Gammaproteobacteria	*Pseudoxanthomonas*	0.08	0.60
	OTU 82	Actinobacteria	*Mycobacterium*	0.00	1.15
	OTU 102	Betaproteobacteria	Unclassified Comamonadaceae	0.03	0.30
	OTU 273	Gammaproteobacteria	Unclassified Gammaproteobacteria	0.04	0.06
	OTU 244	Actinobacteria	Unclassified Actinomycetales	0.15	0.00

*The numbers show average abundance for each OTU in each treatment*.

## Author contributions

SS is a postdoctoral fellow and contributed to qPCR analysis, amplicon data analysis and preparation of the manuscript. MA contributed to the qPCR analysis. ED and NC are the PIs and were responsible for experimental design. ED also contributed to the writing of the paper. AdM contributed to experimental design, set up the microcosm experiment, carried out the amplicon sequencing and the majority of the data analysis and contributed to preparation of the manuscript.

### Conflict of interest statement

The authors declare that the research was conducted in the absence of any commercial or financial relationships that could be construed as a potential conflict of interest.
